# Silencing Stem Cell Factor Gene in Fibroblasts to Regulate Paracrine Factor Productions and Enhance c-Kit Expression in Melanocytes on Melanogenesis

**DOI:** 10.3390/ijms19051475

**Published:** 2018-05-16

**Authors:** Pin-Hui Li, Li-Heng Liu, Cheng-Chung Chang, Rong Gao, Chung-Hang Leung, Dik-Lung Ma, Hui-Min David Wang

**Affiliations:** 1 Department of Fragrance and Cosmetic Science, Kaohsiung Medical University, Kaohsiung City 807, Taiwan; s27159@gmail.com; 2 Department of Life Science, National Chung Hsing University, Taichung City 402, Taiwan; arthur851213@gmail.com; 3 Graduate Institute of Biomedical Engineering, National Chung Hsing University, Taichung City 402, Taiwan; ccchang555@dragon.nchu.edu.tw; 4 Jiaxing Deqin Biotechnology Department, Yangtze Delta Region Institute of Tsinghua University, Jiaxing 314006, Zhejiang, China; gaorong709@naver.com; 5 State Key Laboratory of Quality Research in Chinese Medicine, Institute of Chinese Medical Sciences, University of Macau, Taipa, Macau 999078, China; duncanleung@umac.mo; 6 Department of Chemistry, Hong Kong Baptist University, Kowloon Tong, Hong Kong 22100, China; edmondma@hkbu.edu.hk; 7 Center for Stem Cell Research, Kaohsiung Medical University, Kaohsiung City 807, Taiwan; 8 College of Oceanology and Food Science, Quanzhou Normal University, Quanzhou 362000, China

**Keywords:** stem cell factor, fibroblasts, paracrine factors, melanin, melanocytes

## Abstract

Melanogenesis is a complex physiological mechanism involving various paracrine factors. Skin cells such as keratinocytes, fibroblasts, and melanocytes communicate with one another through secreted regulators, thereby regulating the melanocytes’ bio-functions. The stem cell factor (SCF) is a paracrine factor produced by fibroblasts, and its receptor, c-kit, is expressed on melanocytes. Binding of SCF to c-kit activates autophosphorylation and tyrosine kinase to switch on its signal transmission. *SCF* inhibition does not suppress fibroblast proliferation in MTT assay, and *SCF* silencing induced mRNA expressions of paracrine factor genes, *HGF*, *NRG-1*, and *CRH* in qPCR results. Following UVB stimulation, gene expressions of *HGF*, *NRG*, and *CRH* were higher than homeostasis; in particular, *HGF* exhibited the highest correlation with *SCF* variations. We detected fibroblasts regulated *SCF* in an autocrine-dependent manner, and the conditioned medium obtained from fibroblast culture was applied to treat melanocytes. Melanogenesis-related genes, *tyrosinase* and *pmel17*, were upregulated under conditioned mediums with *SCF* silencing and exposed to UVB treatments. Melanin quantities in the melanocytes had clearly increased in the pigment content assay. In conclusion, *SCF* silencing causes variations in both fibroblast paracrine factors and melanocyte melanogenesis, and the differences in gene expressions were observed following UVB exposure.

## 1. Introduction

The human skin is the largest organ covering the entire body and is made up of three layers, the epidermis, dermis, and subcutaneous layer [1]. The epidermis is distinguished by five layers—the stratum corneum, stratum lucidum, stratum granulosum, stratum spinosum, and the stratum basale (stratum germinativum), from its exterior to the interior. The basal cells in the stratum basale divide continuously, and move toward the stratum corneum [2]. Besides the keratinocytes, the epidermis contains other cells, such as Merkel cells, Langerhans cells, and melanocytes. The dermis is made up of connective tissue and it contains some extracellular matrix components, such as glycosaminoglycan and hyaluronic acid. The dermis possesses sweat glands, sebaceous glands [1,3,4], hair roots, nerves, lymph, and blood vessels. It contains three major cells—the fibroblasts, mast cells, and macrophages. There is a subcutaneous layer under the dermis containing several adipose cells that store fat and maintain body temperature [5]. The skin has certain physiological functions, including protection from pathogens and the external environment, perception of pain or sentience, synthesis of vitamin D, temperature adjustment, absorption, and water resistance [6].

Paracrine factors connect between keratinocytes, fibroblasts, and melanocytes within the skin and play a vital role in ultraviolet (UV)-induced pigmentation and melanocyte activity. More recently, dermal fibroblasts were demonstrated to regulate cutaneous melanin production through secrevarious cytokines. In dark human skin, neuregulin *(NRG)-1* is highly expressed by fibroblasts, suggesting its potential role in constitutive human skin color regulation. Human palmoplantar area is thicker and lighter in color than the nonpalmoplantar area. It has been demonstrated that fibroblasts produce abundant Dikkopf-1 (DKK-1) in the palms and soles, and then suppress the growth and function of melanocytes by arresting the Wnt-signaling pathway [7,8]. In addition, fibroblasts also secrete numerous melanogenic factors, such as stem cell factor (SCF), hepatocyte growth factor (HGF), keratinocyte growth factor (KGF), corticotropin-releasing hormone (CRH), endothelin-1 (ET-1), interferon-γ (IFN-γ), and interleukin-1 (IL-1) [9,10,11,12,13]. Several studies have suggested dermal fibroblasts play a central role in regulating melanocyte functions and influencing human skin pigmentation and constitutive color regulation.

The stratum basale layer of the epidermis, hair, and iris contain a type of cells with the ability to produce melanin for the human body, which are known as melanocytes. Melanocytes are derived from embryonic cells and neural crest cells (NCC), and undergo several life cycles involving melanocyte differentiation from NCC into melanoblasts, the precursor cells. Melanocytes are reported to migrations and proliferations to the target site, differentiations and maturations into melanocytes, transportations and releases of melanosomes filled with melanin to keratinocytes, and cell death [14]. The skin suffers some stresses such as inflammation and free radical accumulation, especially the UV effect on melanin production for defense against injury from the external or internal environment [15]. Melanogenesis is a complex mechanism and process involving intercommunication between melanocytes, keratinocytes, and fibroblasts, thereby regulating signal transduction by secreting paracrine factors and cytokines. There are numerous paracrine factors which regulate melanin production, such as the proopiomelanocortin (POMC)-derived hormone, [(α-MSH and adrenocorticotropic hormone (ACTH)], ET-1, HGF, CRH, nitric oxide, and some inflammatory cytokines (IL-1β, -6, and -10, and TNF-α) [6,16]. Most paracrine factors modulate melanogenesis by upregulating or downregulating the expression of the microphthalmia-associated transcription factor (MITF), which ultimately is the primary transcription factor for tyrosinase, tyrosinase-related protein-1 (TRP-1), and dopachrome tautomerase (tyrosinase related protein-2, TRP-2). Melanin synthesis is accompanied by melanosome maturation, the special cellular organelles in melanocytes. Tyrosinase plays a role as a central and rate-limiting enzyme because it initiates the reaction of tyrosine hydroxylation to L-3,4-dihydroxyphenylalanine (L-DOPA) and oxidization into DOPA quinone. In the presence of thiols such as cysteine or glutathione, DOPA quinone reacts with it and generates cysteinyl-DOPA, turning it into yellow-red pheomelanin. On the other hand, spontaneous cyclization of DOPA quinone to DOPA chrome occurs, followed by a spontaneous loss of carboxylic acid to form 5,6-dihydroxyindole (DHI). The dark-brown eumelanin is converted into indole-5,6-quinone by DHI oxidization via TRP-1 and polymerization. If the DOPA chrome is catalyzed by TRP-2 to DHI-2-carboxylic acid and then oxidized to form indole-5,6-quinone-2-carboxylic acid, its polymerization results in lighter brown eumelanin [17]. The final step of skin pigmentation is the transport of mature melanosomes from melanocytes along with cytoskeletal elements to dendrites, followed by internalization by adjacent keratinocytes contacting with melanocytes [18,19], and 30–40 associated keratinocytes. The mature melanosomes are transported to the adjacent [20,21].

SCF is a paracrine cytokine produced by fibroblasts, keratinocytes, and endothelial cells [22]. *SCF* and its receptor c-kit play an essential role in hemopoiesis and maintaining hematopoietic stem cell survival, mast cell stimulation, mediating the function of the pancreas, and melanogenesis [23]. A mutation in *SCF* could cause anemia, lack of mast cells, immaturity and loss of functions of pancreatic endocrine cells, and disappearance of pigmentation, as well as the absence of melanocytes [24,25]. c-Kit is a receptor tyrosine kinase expressed on melanocytes. It has two domains containing a glycosylated extracellular ligand-binding domain, comprising five Ig-like domains, and a cytoplasmic region, which comprises the protein tyrosine kinase domain. At the ectodomain, five Ig-like domains are separated from the first three domains, constituting a ligand-binding area, and two other domains act on monomerization or dimerization of c-kit. Another part, the phosphorylation site, is situated in the intracellular region, consisting of the juxtamembrane area and a large kinase-insert region [26]. Binding of SCF to c-kit stimulates dimerization and initiation of catalytic activities of autophosphorylation and tyrosine kinase to complete its signal transmission. Y721 phosphorylation of c-kit is involved in the phosphoinositide 3-kinase (PI3K) pathway. PI3K does not only regulate cell survival but also cause pigmentation by increasing the serine/threonine-specific protein kinase AKT activity, which can produce phosphorylate glycogen synthase kinase 3β (GSK-3β) and lead to β-catenin accumulation, resulting in translocation to the nucleus to increase MITF activity [27]. c-Kit phosphorylation at Y703 and Y936 activates the mitogen-activated protein kinase (MAPK) pathway and leads to cell proliferation, differentiation, and melanin alteration [28]. The activated extracellular signal-related kinase *ERK* and c-Jun N-terminal kinase *JNK*, the members of the MAPK family, upregulate the transcription activity or ubiquitin-dependent degradation of MITF as a feedback mechanism of melanin production. On the other hand, the activated *p38* induces phosphorylation of CREB and then activates *MITF* to promote tyrosinase transcription [29]. The other phosphorylation sites such as Y586, Y570, Y900, Y568, Y570, and Y730 are involved in cell proliferation, survival, adhesion, and differentiation, and Y586, Y570, and Y936 participate in receptor downregulation. Some studies even indicated clearly increased expression of *SCF* was observed after UV light exposure, especially UVB, in both keratinocytes and fibroblasts, which facilitated melanogenesis behavior [13,30]. In addition, *SCF* is released in greater levels by fibroblasts than by keratinocytes [31]. Several studies have demonstrated the functions of *SCF*, irrespective of its secretion by keratinocytes or fibroblasts, in proliferation, differentiation, and melanogenesis of melanocytes; however, the importance of *SCF* and its effect on other paracrine factors are yet to be determined [32,33]. Furthermore, fibroblasts play an important role in regulating skin color, and the expression level of *SCF* in fibroblasts is more than that in keratinocytes. Therefore, it is of particular interest to understand the influence on *SCF* in terms of whether its dysfunction is occurring in fibroblasts and then the alteration of other cytokines produced by fibroblasts, as well as melanogenesis in melanocytes. This study examined whether *SCF* silencing influences other paracrine factors secreted by fibroblasts and the changes in melanogenesis of melanocytes.

## 2. Results

### 2.1. The Validation of Short Interfering RNA Knockdown Efficiency

**Figure 1:**
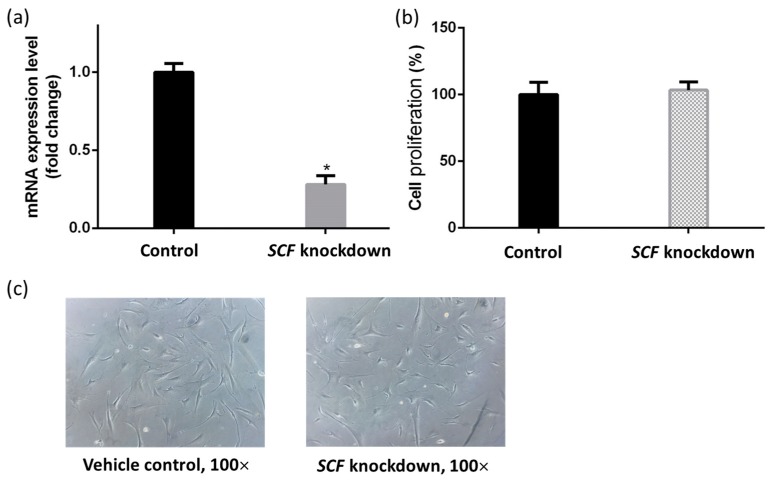
Validation of fibroblasts after *SCF* targeted siRNA 25 nM transfection for 48 h. (**a**) The 75% gene knockdown efficiency of *SCF* in fibroblasts; (**b**) Fibroblasts were treated with 25 nM *SCF*-targeted siRNA for 48 h, and the proliferation was assessed by MTT assay; (**c**) Phase-contrast images compared between control and *SCF* gene knockdown fibroblasts (100×). The data are presented as means ± SD; *n* = 3, * *p* < 0.05.

Transfection was used to introduce siRNA into human fibroblasts. In the attempt to knockdown *SCF* gene expression, it was necessary to validate how successful its genetic knockdown was. Comparisons between the controlled, regular fibroblast cells, and the same fibroblast cells with introduced 25 nM siRNA delineated the productive knockdown in the mRNA expression levels. Figure 1 shows the qPCR-validated knockdown efficiency of *SCF* mRNA levels. qPCR is a quantification technique used to determine the mRNA expression levels of, in this case, the amplified *SCF* gene. Transfection of Hs68 human fibroblasts with 25 nM SCF siRNA showed a significant decrease in *SCF* gene expression 48 h after the transfection compared with regular Hs68 fibroblasts. Using the transfection method and the qPCR confirmation, the results indicated that a successful >75% of *SCF* mRNA expression levels were effectively silenced in Hs68 cells.

### 2.2. The Proliferation of Fibroblasts Was Not Suppressed by SCF Gene Silencing

We next examined if *SCF* inhibited by siRNA caused increased or decreased proliferation of fibroblasts. The MTT method was used to demonstrate fibroblast proliferation. Compared with the vehicle control group, the cell viability showed no difference between the control (100%) and *SCF* gene knockdown fibroblasts (103%). Figure 1b demonstrates neither cellular morphology nor cell confluency were altered. Similar results were observed regarding fibroblast morphology, where without cellular atrophy damage, flattened or low confluency were observed in Figure 1c. The data indicated the lack of an *SCF* gene did not affect cell growth and cellular morphology.

### 2.3. The Variations in Paracrine Factor Expressions through SCF Silencing in Fibroblasts

**Figure 2:**
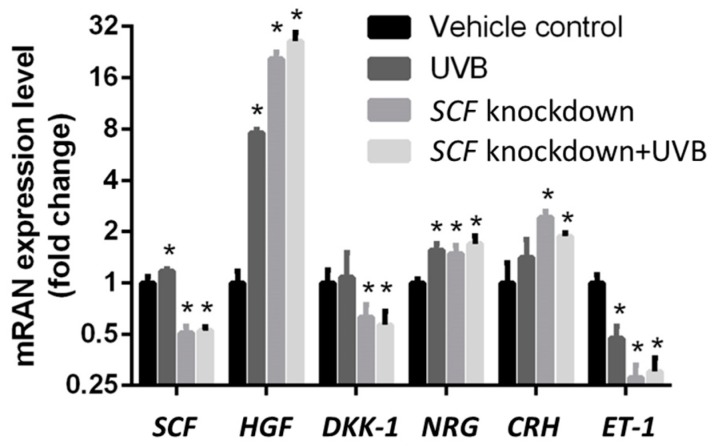
The influences on paracrine factors secreted by fibroblasts were compared between the vehicle control, UVB only, *SCF* knockdown, and *SCF* knockdown with UVB exposure groups. The data are presented as means ± SD; *n* = 3, * *p* < 0.05.

We evaluated whether *SCF* affected the fibroblasts and the mRNA paracrine factor levels from fibroblasts. To determine how differently the paracrine factors reacted due to *SCF* silencing, the controlled gene expressions were compared to the gene expression with SCF knockdown. In Figure 2, the data showed a significant increase in *HGF*, *NRG*, and *CRH* genes when *SCF* was stably silenced 48 h post transfection. The downregulated mRNA levels with *DKK-1* and *ET-1* were affected because of *SCF* knockdown in fibroblast cells. *ET-1* was the most severely affected gene, while the paracrine growth factor *HGF* was the least affected of all the genes. *SCF* gene alteration affected the fibroblast paracrine system and its knockdown increased or decreased all the specific paracrine gene expression levels.

### 2.4. The Variations in Paracrine Factor Expressions through SCF Silencing in Fibroblasts Using the UVB-Stimulated Model

Paracrine factors showed alterations in their mRNA expression levels when the *SCF* gene expression was inhibited. Similar experiments under identical conditions were conducted again with the addition of UVB exposure. Exposure to UVB is known to induce expression of *SCF*, so its effect on mRNA levels compared to the levels without UVB exposure would be a necessary observation. Comparisons between the paracrine factors with and without *SCF* knockdown, and with or without UVB, emphasize the individual gene expression variations not only in terms of *SCF* differences but also in determining whether UVB causes certain factors to react and express differently. In Figure 2, the results show the upregulation gene expressions of *SCF*, *HGF*, *NRG*, and *CRH* with UVB exposure to regular fibroblasts compared with the control group, which did not undergo UVB stimulation. The *ET-1* gene was downregulated, and no significant difference was observed with *DKK-1*. Following the determination of the influence of UVB on regular fibroblasts, the different outcome in fibroblasts with *SCF* gene knockdown under UVB stimulation was tested. A comparison between regular fibroblasts with UVB treatment and *SCF* gene knockdown fibroblasts with the same UVB treatment is presented. The data presented the upregulation mRNA expression of *HGF*, *NRG*, and *CRH* and their expression levels when the fibroblast cells, specifically with stable *SCF* silencing, were exposed to UVB. Compared to the regular fibroblast gene expressions with UVB exposure, *HGF*, *CRH*, and *NRG* with *SCF* knockdowns all had their mRNA expressions increased, even with the same UVB treated situation. HGF demonstrated a particularly dramatic increase in gene expression levels in cells with *SCF* genetic knockdown, while *DKK-1* and *ET-1* were the SCF-silenced genes that were downregulated when treated with UVB. *SCF* gene alteration influenced the fibroblast paracrine system and the condition differed with or without UVB stimulation.

### 2.5. The Influence on Melanocytes When Treated with Conditioned Medium

**Figure 3:**
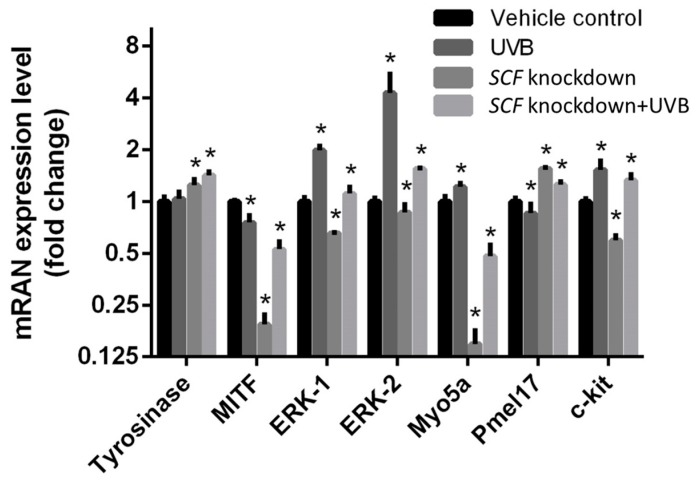
The effects of different conditioned media on the mRNA expression levels were compared with regular fibroblasts, fibroblasts simulated UVB, fibroblasts with SCF knockdown, and fibroblasts exposed UVB with *SCF* gene silenced. The data are presented as means ± SD; *n* = 3, * *p* < 0.05.

To examine the changes in melanocytes caused by fibroblasts with *SCF* gene alteration, the genes related to melanogenesis were analyzed. After the fibroblast cells with or without *SCF* gene were treated with or without UVB, the conditioned medium was collected after 24 h culture and then used to treat melanocytes for 24 h. The experimental conditioned medium obtained from fibroblasts was divided into four experimental groups: fibroblasts without both UVB exposure and *SCF* inhibition, with only UVB exposure, with only *SCF* inhibition, and with both UVB exposure and *SCF* inhibition. These secretions were used as culturing mediums for melanocytes to analyze their different expression levels when cultured in mediums with *SCF* or without *SCF*. In Figure 3, *SCF* gene knockdown, the two conditioned medium groups including the one from regular fibroblasts and *SCF*-silenced fibroblasts, were used to treat melanocytes separately. The gene expression level of tyrosinase and *Pmel17* increased, while *MITF*, *ERK-1*, *ERK-2*, *myosin Va (Myo5a)*, and c-kit decreased. The genes of tyrosinase, *ERK-1*, *ERK-2*, *Myo5a*, and *c-kit* were upregulated when treated in the conditioned medium with UVB compared with the homeostatic condition. In both treatments of UVB exposure with SCF inhibition group, the data showed the alterations in gene expressions in melanocytes when the conditioned medium was used for treatment. Among them, tyrosinase, *MITF*, *ERK-1*, *ERK-2*, *Myo5a*, and *c-kit* were upregulated, and *Pmel17* was downregulated when the conditioned medium from fibroblasts undergoing UVB exposure and SCF silencing was applied to treat melanocytes, compared with the same medium with only UVB stimulation. These findings indicated *SCF* silencing caused the variations in melanocytes and regulated the melanogenesis-related gene expression using the conditioned medium treatment method.

### 2.6. The Variation in Melanin Content in Melanocytes

**Figure 4:**
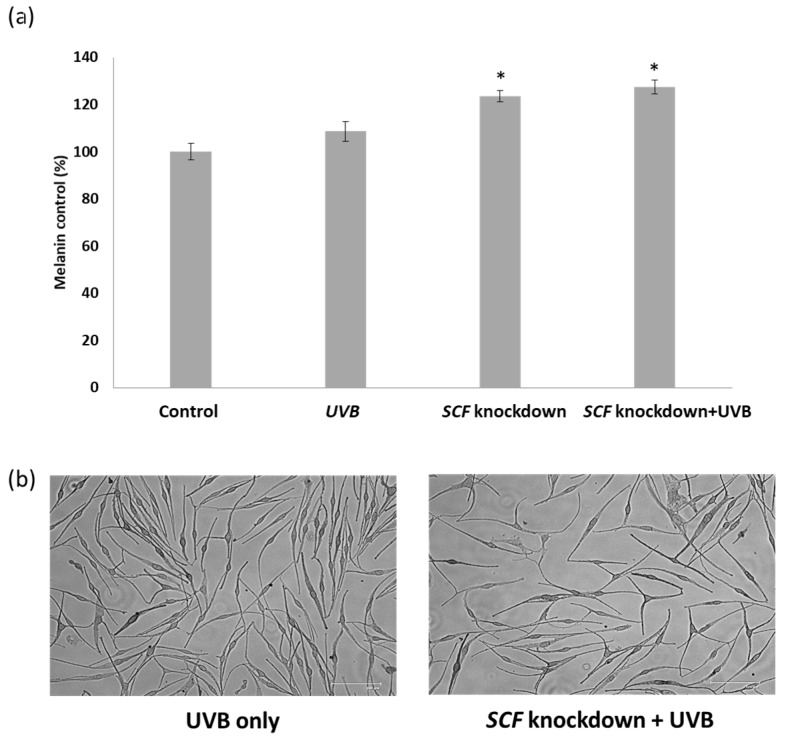
(**a**) The melanin content in melanocytes after being treated with conditioned medium from fibroblast cells for 24 h; (**b**) The morphology of melanocytes was altered (bar = 125 μm). Red arrows showed the melanocytes with dendricity shape morphology. The data are presented as means ± SD; *n* = 3, * *p* < 0.05.

After culturing the melanocyte cells in the conditioned mediums containing the fibroblast cell secretions, the foreign medium could potentially have affected melanocytes and melanin production. The alterations in melanin quantities in the melanocytes were confirmed using the melanin content method. Fibroblasts with exposure to UVB, *SCF* gene knockdown, and *SCF* gene knockdown with exposure to UVB were compared to figure out the actual variation in melanin content in melanocytes due to the different culturing mediums. In Figure 4a, the melanin production in melanocytes increased by 23.5% when treated in the conditioned medium from *SCF* fibroblasts knockdown compared to the normal fibroblasts and increased 27.3% under *SCF* fibroblasts knockdown with UVB exposure. In Figure 4b, the morphology of melanocytes was altered by treatment with the conditioned medium of fibroblasts with *SCF* knockdown and UVB exposure, resulting in the melanocytes getting more synapses than UVB only. The result was consistent with Figure 2, indicating *SCF* silencing caused an essential variation in melanin quantities in the melanocytes.

### 2.7. c-Kit Activation and SCF Regulation through Autocrine Mechanism

**Figure 5:**
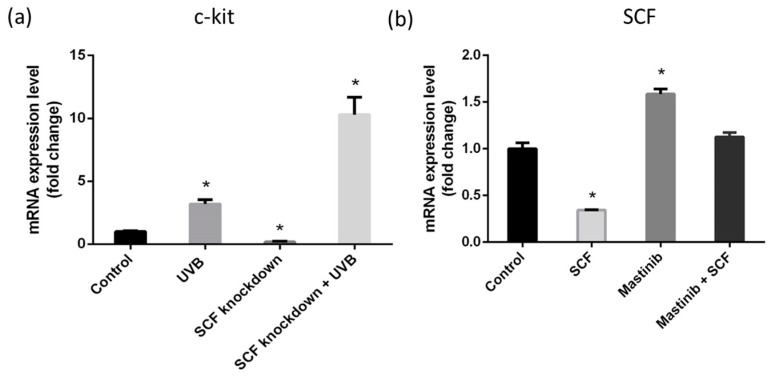
Autocrine of SCF on fibroblasts. (**a**) The expression of c-kit on fibroblast; (**b**) Fibroblasts treated with SCF 30 ng, mastinib 3 μM, and both of mastinib and SCF. The data are presented as means ± SD; *n* = 3. * *p* < 0.015.

Using qPCR, we observed fibroblasts had a c-kit receptor that changed the expression level under different conditions. Figure 5a shows c-kit gene expression was increased with UVB stimulation, even under *SCF* gene silencing by siRNA transfection compared with regular fibroblasts. In an attempt to demonstrate the existence of an autocrine system in fibroblast cells, different tests were conducted. In Figure 5b, the fibroblast’s inner SCF levels were downregulated when treated with 30 ng of recombinant *SCF*, while the fibroblast’s inner SCF levels were upregulated when treated with 3 µM of masitinib, which is an inhibitor of *SCF* and c-kit. When combined, 30 ng of SCF and 3 µM of masitinib produced a result similar to the controlled fibroblast’s inner SCF level. When treated with inhibiting or facilitative SCF environments, fibroblast cells varied their inner SCF levels to balance out their inner and outer levels, confirming the existence of an autocrine signaling system.

## 3. Discussion and Conclusions

In human skin, melanosomes are released from melanocyte dendrites and taken directly up by keratinocytes through endocytosis or phagocytosis. Little was known concerning the relationships between the morphology and the melanin content or tyrosinase activities of the melanocytes. In previous keratinocyte and melanocyte co-culture studies, we found that as melanocytes lose their characteristic dendritic structures and adopt fibroblast-like bipolar forms, the cell–cell contact between melanocytes and keratinocytes is considerably reduced, resulting in a reduction in pigment transfer [34]. Our previous two studies suggested that there might be some connections between the dendritic morphology changes and physiological properties of melanocytes [12,33]. These potential relationships should be further investigated to understand their physiological significances.

SCF is a paracrine cytokine that plays an essential role in hematopoiesis, maintenance of cell survival, proliferation, and activation, as well as in melanogenesis. Although several studies demonstrated the functions and mechanisms of SCF in the melanogenesis process, those studies mostly focused on pigment production only. This study examined the role of SCF in paracrine factors in fibroblasts and its subsequent influence on melanocytes under a loss of SCF function in fibroblasts. RNA interference (RNAi) is a physiologically mediated mechanism that induces sequence-specific gene degradation. The double-strand RNA undergoes the dicer processes into the siRNA. Binding of siRNA to the target gene causes mRNA degradation directly or arrests the specific protein translation; therefore, the phenomenon is known as gene silencing [35]. Consequently, siRNA is suitable for observing the gene influences, protein functions, and cell developments when the target gene is knocked down and for understanding the role of the target gene. This study used the *SCF*-targeted siRNA-cooperated transfection method to introduce siRNA into the intracellular environment and inhibit *SCF* gene expression in fibroblasts. In this study, all the experiments processed the validation and kept the transfection efficiency at a stable inhibitory state.

After siRNA transfected into the intracellular environment and induced cellular *SCF* gene inhibition, the first aspect to confirm was whether the gene silencing caused cell death or a morphological change, since SCF was responsible for cell survival and proliferation. In MTT assay data, it presented no significant changes in fibroblast cell proliferations, and similar results were observed regarding fibroblast morphology, where without cellular atrophy damage, flattened or low confluency were observed. Fibroblast proliferation did not rely on *SCF*, even though it was silenced. Although *SCF* inhibition did not cause fibroblast death, the influence on fibroblasts was unclear. To explore the effect on fibroblasts when the *SCF* gene was silenced, some paracrine factors secreted by fibroblasts showing effects on melanocyte cell survival and melanin production were determined, including *HGF*, *DKK-1*, *NRG-1*, *CRH*, and *ET-1*.

HGF is a polypeptide growth factor that can bind to the MET receptor expressed on melanocytes, with HGF stimulation leading to MITF-dependent Met message and protein induction [36]. The MET proto-oncogene encodes for the hepatocyte growth factor (HGF) receptor, a plasma membrane tyrosine kinase that is involved in melanocyte growth and melanoma development. When the ligand binds to the receptor, MET processes autophosphorylation on tyrosine residues produce the docking site for PI3K to promote growth and differentiation of melanocytes, thereby influencing melanogenesis via activation of the cyclic adenosine monophosphate (cAMP) pathway [37]. DKK-1 secreted by fibroblasts mostly exists in the palmoplantar skin compared with the nonpalmoplantar skin [38]. DKK-1 has the ability to inhibit melanocyte growth via regulating the Wnt signaling pathway in which DKK-1 suppresses β-catenin accumulation and then decreases MITF activity, thereby influencing the functions of the melanocytes. In a previous study, Wonseon Choi et al. used the microarray analysis to indicate in dark skin, and NRG-1 was the highly expressed factor secreted by fibroblasts and increased skin pigmentation [6]. NRG-1 mediates melanogenesis through the PI3K pathway, increasing melanocyte proliferation and survival and melanin production. CRH is regulated by UV or as a response to stress. CRH can bind to its receptors, CRH-R1 and CRH-R2, and then induce the POMC-derived hormones, including MSH and ACTH released to influence the skin phenotype system [39]. ET-1 functions as a mitogenic factor for melanocytes and facilitates melanogenesis. ET-1 can bind to its endothelin receptors A (ETA) and activate the cAMP pathway that increases protein kinase C activation to stimulate MITF expression. Akira Hachiya et al. demonstrated the responses of SCF and ET-1 to UVB irradiation, in which SCF responded in the early stage and ET-1 in the later phase of UVB-induced melanin production [40,41].

Through *SCF*-targeted siRNA inhibition, we demonstrated *HGF*, *NRG*, and *CRH* gene expressions were significantly increased; especially, *HGF* gene level had the highest change while *DKK-1* and *ET-1* gene expressions were decreased. The results demonstrated an autoregulation phenomenon between gene networks, inducing a feedback expression when the *SCF* gene was silenced in fibroblasts. Cellular autoregulation is frequently observed in gene networks through either negative or positive feedback adjustments [42]. Among them, *HGF* showed 20-fold high expression in fibroblasts after *SCF* gene silencing compared with regular fibroblasts. Some experiments demonstrated a similar result that SCF expressed in murine stromal cells was activated and induced an increased expression when treated with extra HGF [43,44,45]. This demonstration indicated the relationship between *HGF* and *SCF*. Hence, in this case, *SCF* gene knockdown induced transcription of *HGF* for facilitating the expression of *SCF* to balance the gene networks. *NRG* and *CRH* involved in melanogenesis also increased their gene expressions under the condition of *SCF* gene knockdown. On the other hand, *DKK-1*, a Wnt-antagonist, was downregulated after *SCF* was inhibited. Gherghe et al. indicated Wnt treatment significantly activated *HGF* expression in human endothelial progenitor cells [46]. In this case, the Wnt-antagonist *DKK-1* was downregulated to avoid the Wnt signaling system inhibition, thereby increasing the expression of *HGF*. The other downregulated gene, *ET-1*, was indicated for the mechanism of synergistic effect on *ET-1* and *SCF* that led to melanogenesis. Therefore, *SCF* inhibition influences *ET-1* gene expression.

Under the homeostatic status, some paracrine factors are changed or their expression is induced by cells suffering from different stresses such as UV. *SCF* plays a potential role in regulating melanogenesis under homeostatic and stimulatory statuses, especially in response to UVB irradiation [47]. It is known that UVB upregulates transcription, increases the expressions of *c-kit* and *SCF*, and stimulates melanogenic activity in melanocytes. To understand whether the stimulation with UVB when *SCF* was silenced would influence the paracrine factors in fibroblasts, an experiment with gene knockdown and then treatment with UVB were performed. The results showed fibroblasts under UVB exposure increase the mRNA expression levels of *SCF*, *HGF*, *DKK-1*, *NRG*, and *CRH* related to the melanogenesis gene. Under the same condition, *ET-1* was downregulated, as shown in previous studies that reported *ET-1* was responsible for a later stage of UVB-induced melanin production. Then, comparison between only UVB-treated and both *SCF* gene knockdown and UVB-stimulated fibroblasts showed similar results but increasing levels of *HGF*, *NRG*, and *CRH* gene expressions under an *SCF*-silenced status. Among them, *HGF* showed more than a 3-fold change compared to only UVB-treated fibroblasts and more than a 20-fold change compared to regular fibroblasts. It was demonstrated *SCF* was important for UVB-induced paracrine secretion, so *HGF* needed to strengthen its expression to balance the defect from *SCF* silencing. In addition, *SCF* and *HGF* showed a high correlation in fibroblasts with either UVB stimulation or the homeostatic condition [48,49].

Silencing *SCF* affected paracrine factor expressions in the fibroblasts. Melanocytes receive the signals generated from fibroblasts and then activate melanin production. In subsequent experiments, we investigated whether *SCF*-silenced fibroblasts further influenced melanocytes for melanogenesis, and the gene expression in melanocytes was determined. Melanin biosynthesis is a complex process occurring in melanocytes within particular membrane-bound organelles, known as melanosomes [39]. The major component of the melanosome is *Pmel17*, which is a transmembrane protein and essential for melanin synthesis and deposition to form the fibrillar matrix. Rab27a, melanophilin (MLPH), and Myo5a form a tri-protein complex to bind melanosomes at the melanocytes peripheries. In the process of melanosome transport, the ternary complex is the connection between actin cytoskeleton and melanosome. A lack of these proteins affects the transport, and melanoregulin (Mreg) drives melanosome transfer from melanocytes to keratinocytes via a regulated shedding mechanism. Human skin melanin is driven by the intercellular movement of melanin-containing melanosomes from the extremities of human melanocytes dendrites to neighboring keratinocytes. When it is carried by the actin filament, melanosome moves to the dendritic tail section, through exocytosis, and is transported into keratinocytes [50]. The greater the amount of melanin that is transferred into keratinocytes, the darker is the color of the skin [18]. The movement on the microtubule depends on the dynein–dynactin motor complex. Mreg forms a complex with Rab-interacting lysosomal protein and p150 (Glued), which is a subunit of dynactin [51]. Mreg adjusts a shedding system which makes melanosome transport from human melanocytes to keratinocytes. The shedding process from human melanocytes of melanosome-rich packages is undergoing the phagocytosis of keratinocytes. The shedding not only takes place principally at dendritic extremities, but also around the center areas, having the adhesion to keratinocytes, tightening behind the forming packages, and apparent self-abscissions [52]. The movement on the actin filament requires Myo5a, Rab27a, and MLPH as the connecting bridge [53]. 36H downregulated protein expression for Myo5a and might prevent a darkening of skin color. Melanin is deposited on these fibers and melanosomes become mature in the melanocytes [54]. Melanocytes are activated through multiple paracrine factors secreted from adjacent cells, such as keratinocytes and fibroblasts. The paracrine factors bind to the specific receptors expressed on melanocytes and then accelerate signal transductions to initiate melanin production. For example, SCF binds to the c-kit receptor, and MAPK family, *ERK1/2*, *JNK*, and *p38*, are activated in the melanocytes, thereby altering MITF activities and functions through phosphorylation or dephosphorylation. MITF is a specific transcription factor of tyrosinase, *TRP-1* and *TRP-2*, and the mutant of MITF leads to a deficiency in melanocytes. Melanin is the end product of the multistep conversion of tyrosine. Tyrosinase, a rate-limiting enzyme, catalyzes tyrosine into DOPA, and subsequently DOPA undergoes a separate pathway to convert to pheomelanin or eumelanin. After maturation of melanin, it is transferred to keratinocytes along with microtubules and dendrites by the kinesin and dynein motor proteins. Actin-based motor protein myosinVa attaches to melanosomes and moves via interaction with Rab27a and melanophilin to the surrounding keratinocytes.

In this work, to avoid direct UVB exposure and for observing the influence from fibroblasts, culture conditioned mediums from fibroblasts were harvested and used to treat melanocytes. Using the conditioned medium from regular fibroblasts and fibroblasts with UVB treatment, *ERK-1*, *ERK-2*, *Myo5a*, and *c-kit* genes were upregulated while MITF and *Pmel17* were downregulated when treated with UVB-exposed FB medium. According to the above results, UVB-induced *SCF*, *HGF*, *NRG*, and *CRH* increased. *ERK-1* and *ERK-2* were significantly increased, attributable to both the *SCF* and *HGF* signaling transduction pathways. UVB induced SCF production, so the receptor *c-kit* expressed on melanocytes increased. In contrast, MITF gene expression was downregulated under UVB-exposed fibroblast medium treatment. McGill et al. indicated MET, the *HGF* receptor, was regulated by MITF, since MET was a transcriptional target of MITF. In this case, *HGF* was increased from the conditioned medium and caused high MET expression in the melanocytes, so MITF decreased to reduce MET overexpression [55].

After treatment with the conditioned medium from fibroblasts with *SCF* silencing, the melanocytes showed downregulation of *MITF*, *ERK-1*, *ERK-2*, *Myo5a*, and *c-kit*, while tyrosinase and *Pmel17* were upregulated, compared with treatment with regular fibroblast medium. Under this situation, *SCF* was inhibited by siRNA, so the expression was followed by a decrease, and then the melanocytes treated with this medium showed decreased gene expression of *ERK-1*, *ERK-2*, and *SCF* receptor *c-kit*. Comparing regular fibroblasts exposed to UVB and SCF-inhibited fibroblasts treated with UVB stimulation, the melanocytes cultured in the conditioned medium showed increased gene expression of tyrosinase, *MITF*, *Myo5a*, and *c-kit*. According to the above results, *HGF*, *NRG*, and *CRH* were upregulated in *SCF*-silenced fibroblasts with UVB exposure compared with fibroblasts treated with only UVB, so the melanocytes accepted more stimulation to increase the melanogenesis-related gene expression level. *SCF* silencing also caused *ERK-1* downregulation, but *ERK-2* showed no significant change in its gene level compared to regular fibroblasts exposed to UVB in melanocytes. In addition, this result is observed when the *SCF* gene is knocked down, the gene *Myo5a* is downregulated, and *Pmel17* is upregulated whether there is UVB exposure or not.

To confirm the essential changes in the melanocyte, the quantity of melanin production was examined. The melanin content assay showed melanocytes cultured with conditioned medium from *SCF*-silenced fibroblasts exposed to UVB increased the amount of melanin by about 18%, compared with regular fibroblasts exposed to UVB. This result was consistent with the abovementioned data that melanogenesis-related genes were upregulated. Based on these findings, indeed, *SCF* silencing caused variations in fibroblasts to paracrine factors, and the variation changed if fibroblasts suffered UVB stimulation. The results from different fibroblasts affected melanocyte activities via melanogenesis-related gene expression. Especially under the condition of fibroblasts with *SCF* silencing and then exposure to UVB, the melanocytes cultured in fibroblast secretions exhibited increased melanogenesis-related gene levels, and the phenomenon was confirmed by their increased melanin quantities [36]. Analysis of the *SCF* receptor *c-kit* mRNA expression level in the fibroblasts showed the *c-kit* gene level increased under *SCF* inhibition in fibroblasts and further increased when fibroblasts with *SCF* inhibition were exposed to UVB. Although *c-kit* showed increased expression following UVB exposure with or without *SCF* gene knockdown, the underlying mechanisms need to be confirmed. A probable reason would be that as *SCF* and *c-kit* were induced by UVB stimulation and *SCF* was knocked down, *c-kit* increased its expression to improve the binding of the ligand to the receptor.

**Figure 6:**
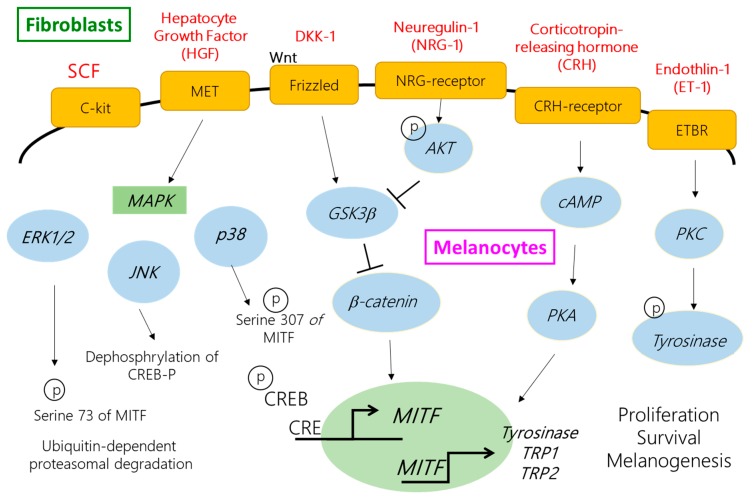
The mRNA pathway of *SCF* in fibroblasts affects the melanogenesis in melanocytes.

Autocrine signaling is a type of cell regulation in which cells secrete and respond to their particular growth factors. According to fibroblast autoregulated gene expression under *SCF* gene knockdown and *c-kit* expression on fibroblasts, the fibroblasts regulated *SCF* produced in an autocrine manner. Addition of recombinant *SCF* resulted in a decreased expression level, showing a natural feedback in that the fibroblast cells stopped producing as much *SCF* [8]. Similarly, adding masitinib inhibited the binding between *SCF* and *c-kit*, and so the expression level was increased. The fact that adding both masitinib and *SCF* produces similar expression levels as the control indicated the automatic balancing of the inner *SCF* levels, which is the role of an autocrine signaling system. A similar SCF autocrine effect was demonstrated in several tumors [56,57]. These results further showed the existence of an autocrine system with co-expression of *SCF* and *c-kit* in fibroblasts. The present study revealed that although *SCF* is involved in several processes such as cellular differentiation, proliferation, and survival, it does not influence fibroblast proliferation. Once *SCF* is silenced in fibroblasts, the autoregulation in gene networks balances the *SCF* defects in an autocrine regulatory manner. Investigation of the paracrine factors HGF, DKK-1, NRG, CRH, and ET-1 showed *HGF* exhibited an especially high correlation with *SCF.* Fibroblasts with *SCF* dysfunction influenced their own paracrine systems and further affected melanocytes to alter melanin production, particularly under UVB stimulation (Figure 6). Altogether, *SCF* plays an essential role in fibroblast paracrine factors and in the melanocyte cellular melanogenesis effect.

## 4. Materials and Methods

### 4.1. Reagents and Materials

Cell culture materials Medium 254, Medium 254 supplement, fetal bovine serum (FBS), Dulbecco’s modified Eagle medium (DMEM), and antibiotics were obtained from Gibco (Waltham, MA, USA). Short interfering RNA (siRNA) transfection reagent, Trizol reagent, 1-bromo-3-chloropropane (BCP), and 3-(4,5-dimethylthiazol-2-yl)-2,5-diphenyltetrazolium bromide (MTT) were purchased from Sigma Chemical (St. Louis, MO, USA). ToolScript MMLV RT kit and TOOLS 2× SYBR quantitative real-time polymerase chain reaction (qPCR) Mix was purchased from Biotools (Taipei, Taiwan).

### 4.2. Cell Cultures

The neonatal foreskin human melanocytes purchased from Cascade Biologics (C-102-5C, Gibco) were maintained in Medium 254 supplemented with human melanocyte growth supplement containing basic FGF (3 ng/mL), insulin (5 µg/mL), transferrin (5 µg/mL), bovine pituitary extract (0.2%), FBS (0.5%), heparin (3 µg/mL), hydrocortisone (0.18 µg/mL), and phorbol 12-myristate 13-acetate (10 ng/mL). The human fibroblasts Hs68 cells derived from male foreskin (ATCC^®^ CRL-1635^™^) were obtained from the Food Industry Research and Development Institute (FIRDI, Hsinchu, Taiwan) and cultured in DMEM supplemented with 10% FBS and 1% antibiotic. All cells were incubated at 37 °C under 5% CO_2_ [8,13].

### 4.3. Transfection of SCF-Targeted siRNA into Fibroblasts

Fibroblasts were seeded in a 6-well plate at a density of 2 × 10^5^ cells and then cultured for 24 h until the cells reached about 80%–90% confluence. Then, the siRNA mixture was prepared for further experiments. SCF-targeted pooled siRNA supplied by Dharmacon (GE Dharmacon, Lafayette, CO, USA) was diluted to 25 nM in 100 µL per well of serum-free DMEM and 20 µL per well transfection reagent was added into the diluted siRNA solution. After mixing and incubation for 20 min, 100 µL of the mixture was added to each well and incubated for 3 days at 37 °C under 5% CO_2_. The control group was fibroblasts transfected reagent without siRNA. The transgene expression efficiency was detected by qPCR. The siRNA sequences used in the experiment are shown in Table 1.

### 4.4. MTT Assay in Cell Viability

MTT assay was used to evaluate cell viability and proliferation. MTT, a tetrazole with a yellow color, is taken up by living cells and then converted into a purple formazan-reduced state by mitochondrial dehydrogenase. Cells were seeded in a 96-well plate at a density of 8 × 10^3^ cells per well and cultured for 24 h. Following attachment, cells were replaced in fresh medium with siRNA and transfection reagent complex and cultured for 48 h. Then, the medium was changed to fresh medium containing 100 µL of 0.5 mg/mL MTT and then cultured for 2 h at 37 °C under 5% CO_2_. Then, the medium was discarded and 100 µL DMSO was added to dissolve the formazan. The absorbance was measured at 595 nm [15].

### 4.5. UVB Exposure to Fibroblasts and Melanocytes Treated with Conditioned Medium

To induce *SCF* secretion from fibroblasts and simulate the condition of light stimulation, UVB was used to complete the test. This test was performed according to the method, with some modifications, described by Shin et al. [13]. Fibroblasts with or without previous siRNA transfection were cultured in a 6-well plate. Before UV exposure, the medium was replaced by PBS during that time. UVB exposure was performed using Ultraviolet Crosslinkers CL-1000 (UVP, Upland, CA, USA) with UVB radiation (302 nm) at a nontoxic dosage of 5 mJ/cm^2^ 3 times at intervals of 5 h. After UVB treatment and culturing for 24 h, the medium was collected into the conditioned medium and used for treating melanocytes previously cultured in the 6-well plate for 24 h. Experimental cells were harvested for subsequent testing.

### 4.6. Total RNA Isolation and Extraction

The total RNA was extracted using Trizol RNA isolation reagent, which can break down cell lysates and isolate RNA, DNA, and proteins. First, 1 mL Trizol reagent was added to each and transferred to a 1.5-mL microtube at room temperature for 5 min. Then, 200 µL BCP per mL of Trizol reagent was added and mixed vigorously. Following incubation for 2 min, the samples were centrifuged at 14,000× *g* for 15 min. The sample homogenates formed two phases, from which the aqueous phase on the top of the homogenate was transferred to a new Eppendorf tube. To precipitate RNA, an equal volume of isopropanol was added and mixed. The mixture was centrifuged at 14,000× *g* for 15 min and the supernatant was removed. The RNA pellet was washed with 1 mL of 75% ethanol to remove the residual salts. Finally, the mixture was centrifuged at 12,000× *g* for 5 min and the RNA pellet was dried and dissolved with 50 µL diethylpyrocarbonate (DEPC)-treated water. The concentration and quality of the RNA extracts were determined by NanoDrop (Thermo Fisher, Waltham, MA, USA).

### 4.7. Detection of mRNA Gene Expression Using qPCR

Before qPCR analysis, RNA should be processed by a reverse transcription step to obtain [8] complementary DNA (cDNA). cDNA synthesis was accomplished by ToolScript MMLV RT kit. Briefly, 1 µg total RNA of each sample was mixed with 2 µL oligo dT, 2 µL dNTP, and distillation-distillation H_2_O to bring the total volume to 14.5 µL. To open the RNA secondary structure, the RNA mixture was heated at 70 °C for 5 min and then cooled on ice for 2 min. To the cooled mixture were added 4 µL reverse buffer, 0.5 µL RNasin, and 1 µL MMLV. Afterward, the mixture was incubated at 42 °C for 50 min to synthesize cDNA and then heated at 95 °C for 5 min to inactivate the MMLV. Further, the cDNA products were processed on qPCR using TOOLS 2× SYBR qPCR Mix. First, a reaction solution was prepared containing 10 µL 2× SYBR qPCR mix, 0.1 µL forward primer, 0.1 µL reverse primer, 1 µL cDNA template, 2 µL of 50× ROX reference dye, and distillation–distillation H_2_O to bring the total volume to 20 µL per well. Next, the qPCR machine (ABI^TM^ StepOne^TM^ Plus, Thermo Fisher) was set up. The initial denaturation temperature was 95 °C for 15 min by one cycle, followed by a denaturation temperature of 95 °C for 10 s, annealing at 60 °C for 20 s, and extension at 72 °C for 30 s, with the total PCR stage being 40 cycles. The designed forward and reverse primers from 5′ to 3′ used in this experiment are shown in (Table 2) [8].

### 4.8. Melanin Content Assessment

Estimation of melanin content is a method for cellular melanin determination. At first, cells were seeded in a 6-well plate at a density of 2 × 10^5^ cells and cultured for 24 h. Then, the medium was changed to conditioned medium obtained from fibroblasts cultured for 24 h. Afterward, the cell pellets were harvested and dissolved in 2.0 N NaOH, and then heated at 95 °C for 1 h. The absorbance of melanin was measured at 490 nm [30].

### 4.9. Statistical Analysis

All the experiments in each platform were carried out in triplicate and presented as mean ± standard error. For statistical analysis, all data were analyzed by Student’s *t*-test for multiple comparisons. A significant difference (*) was defined as *p* < 0.05.

## Figures and Tables

**Table 1:** *SCF*-targeted siRNA sequences used in this study.

Target sequence 1: GGAAUCGUGUGACUAUAA
Target sequence 2: AUAAGUAUGUUGCAAGAGA
Target sequence 3: UAAGCGAGAUGGUAGUACA
Target sequence 4: GCUUUAUAGUUGCCGAUUA

**Table 2:** The primer sequences used in this study.

SCF
Forward: 5′-CTGCCAACGATCCTATCTTCCT-3′
Reverse: 5′-GGTTATGTCCAATGGGTGCATT-3′
HGF
Forward: 5′-TCCCTACCTCTCTCGCTGTCT-3′
Reverse: 5′-GGGTAAGGGCCAGCATGTA-3′
DKK-1
Forward: 5′-GCGGGAATAAGTACCAGACCAT-3′
Reverse: 5′-TGCAGGCGAGACAGATTTG-3′
NRG
Forward: 5′-AGGCCAGGACCCTATTATTTC-3′
Reverse: 5′-TTCAGATTGAGCCCTAGAGACA-3′
CRH
Forward: 5′-AGGCACCGGAGAGAGAAAG-3′
Reverse: 5′-GTTTCCTGTTGCTGTGAGCTT-3′
ET-1
Forward: 5′-TGGTTCCTGACTGGCAAAG-3′
Reverse: 5′-GGAAGCCAGTGAAGATGGTT-3′
Tyrosinase
Forward: 5′-CTGCCAACGATCCTATCTTCCT-3′
Reverse: 5′-GGTTATGTCCAATGGGTGCATT-3′
MITF
Forward: 5′-TTGGTGCCACCTAAAACATTGT-3′
Reverse: 5′-CCGTTGGGCTTGCTGTATG-3′
ERK-1
Forward: 5′-CAACACCACCTGCGACCTT-3′
Reverse: 5′-GCCACATACTCCGTCAGGAA-3′
ERK-2
Forward: 5′-CGGTGTTCTTCTTCCCAGTTC-3′
Reverse: 5′-AAAGCCACAACTACCAGAAACC-3′
Pmel17
Forward: 5′-GGATGGTACAGCCACCTTAAGG-3′
Reverse: 5′-CAGGATCTCGGCACTTTCAATAC-3′
Myo5a
Forward: 5′-GCCCAGATTGTGAAAGTGTTGA-3′
Reverse: 5′-CCTGTCTCGTAAACGCATCTGT-3′
